# *Gentiana lutea* Extract Modulates Ceramide Synthesis in Primary and Psoriasis-Like Keratinocytes

**DOI:** 10.3390/molecules25081832

**Published:** 2020-04-16

**Authors:** Fabian Gendrisch, Anna Nováčková, Michaela Sochorová, Birgit Haarhaus, Kateřina Vávrová, Christoph M. Schempp, Ute Wölfle

**Affiliations:** 1 Department of Dermatology, Medical Center, Faculty of Medicine, University of Freiburg, 79106 Breisgau, Germany; fabian.gendrisch@uniklinik-freiburg.de (F.G.); birgit.haarhaus@uniklinik-freiburg.de (B.H.); christoph.schempp@uniklinik-freiburg.de (C.M.S.); 2 Skin Barrier Research Group, Charles University, Faculty of Pharmacy in Hradec Králové, Akademika Heyrovského 1203, CZ-50005 Hradec Králové, Czech Republic; novacka1@faf.cuni.cz (A.N.); sochorovam01@gmail.com (M.S.); vavrovak@faf.cuni.cz (K.V.)

**Keywords:** ceramides, skin, *Gentiana lutea*, CerS, ELOVL

## Abstract

*Gentiana lutea* is a bitter herb that is traditionally used to improve gastric disorders. Recently, we have shown that *Gentiana lutea* extract (GE) also modulates the lipid metabolism of human keratinocytes in vitro and in vivo. In the present study, we investigated the role of GE on ceramide synthesis in human primary keratinocytes (HPKs) and psoriasis-like keratinocytes. We could demonstrate that GE increased the concentrations of glucosylceramides and the ceramide AS/AdS subclass without affecting the overall ceramide content in HPKs. The expression of ceramide synthase 3 (*CERS3*) and elongases (*ELOVL1* and *4*) was reduced in psoriasis lesions compared to healthy skin. Psoriasis-like HPKs, generated by stimulating HPKs with cytokines that are involved in the pathogenesis of psoriasis (IL-17, TNF-α, IL-22 and IFN-γ) showed increased levels of IL-6, IL-8 and increased expression of *DEFB4A*, as well as decreased expression of *ELOVL4*. The treatment with GE partly rescued the reduced expression of *ELOVL4* in psoriasis-like HPKs and augmented *CERS3* expression. This study has shown that GE modulates ceramide synthesis in keratinocytes. Therefore, GE might be a novel topical treatment for skin diseases with an altered lipid composition such as psoriasis.

## 1. Introduction

The outermost layer of the human skin, the stratum corneum (SC), protects the body against excessive water loss and penetration of undesired substances from the environment [1]. The SC consists of dead cells, corneocytes, filled with keratins and an extracellular lipid matrix. These multiple lipid lamellae are essential for a correct epidermal permeability barrier [2,3] and contain mainly ceramides (approximately 50% of the SC lipids), as well as cholesterol and free fatty acids (FA). Ceramides, which are simple sphingolipids composed of a sphingoid base and FA chain, are produced from their polar precursors (glucosylceramides and sphingomyelins) and delivered to the SC through lamellar bodies. The skin barrier ceramides are a highly heterogeneous lipid class and they differ in their polar head structure, chain length, hydroxylation and esterification pattern [4,5].

Psoriasis is a T cell mediated autoimmune disease characterized by hyperkeratosis [6,7] and epidermal barrier abnormalities including disturbed ceramide synthesis [8]. In psoriasis, the expression of differentiation markers such as filaggrin and keratin 1 is reduced [9]. In addition, ceramide levels are reduced in psoriatic epidermis compared to healthy skin, which correlates with an increased transepidermal water loss (TEWL) [10,11]; reviewed in [12]. The de novo synthesis of ceramides also negatively correlates with the clinical severity of psoriasis [13]. Enzymes that generate long chain ceramides, elongases (ELOVLs) and ceramide synthases (CerS), are reduced in psoriatic skin [14,15]. In addition, it was shown that ceramides NH and NP with very long FAs (longer than C26) are decreased in psoriasis, whereas ceramide NS with shorter FAs (shorter than C24) are increased [15]. This overall decrease in ceramide chain lengths also contributes to the barrier abnormality in psoriasis [16], as the average FA chain length of ceramides correlates with the barrier function of the epidermis [16]. Tawada and colleagues also showed that ELOVL4 and ceramide synthase 3 (CERS3) expression is decreased in IFN-γ stimulated keratinocytes [16] and IFN-γ has been shown to be highly expressed in skin lesions of psoriasis patients and might be partially associated with barrier dysfunction in psoriasis [17]. 

Such skin barrier defects may lead to the development of epidermal hyperplasia and inflammation [18] and the exacerbation of psoriasis. Thus, using drugs that stimulate the expression of ELOVLs and CERS3 could be beneficial for patients with psoriasis [17].

*Gentiana lutea* L. is an herbal bitter drug that improves gastric disorders. Recently, we have shown that *Gentiana lutea* extract (GE) and amarogentin stimulate the synthesis of epidermal barrier proteins (e.g., keratins) and lipids in vitro and in vivo [19].

In the present study, we wondered whether GE modulates the synthesis of ceramides (or sphingolipids in general) in keratinocytes, in particular through modulating the expression of enzymes involved in the generation of long chain ceramides (ELOVLs, CerS). We first studied the levels of ceramides, glucosylceramides and sphingomyelins in human primary keratinocytes (HPKs) and verified the GE effects on sphingolipid metabolism. Then, the levels of ceramides and glucosylceramides, as well as the protein expression of ELOVLs and CerS3, in the lesional skin of patients with psoriasis and healthy volunteers were investigated. Finally, we generated psoriasis-like keratinocytes by treating HPKs with cytokines that are involved in the pathogenesis of psoriasis (IL-17, TNF-α, IL-22 and IFN-γ), and studied the effects of GE treatment on the expression of *ELOVL*s and *CERS3*.

## 2. Results

### 2.1. Impact of GE on Ceramide Concentration in Human Keratinocytes 

**Figure 1:**
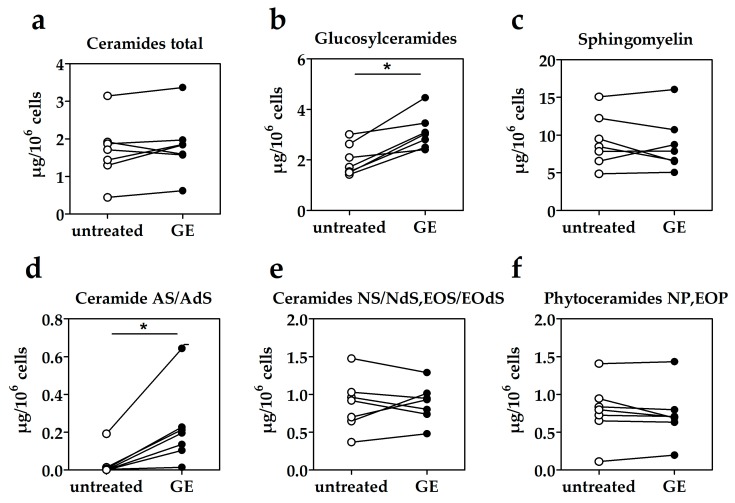
Effects of *Gentiana lutea* extract (GE) treatment on the concentrations of ceraimides and their precursors (glucosylceramides and sphingomyelins) in human primary keratinocytes (HPKs). HPKs were either treated for 1 week with GE (200 µg) or left untreated. (**a**)–(**f**) Lipids were analyzed by high performance thin layer chromatography (HPTLC). * *p* ≤ 0.05.

First, we analyzed if the concentrations of ceramides, glucosylceramides and sphingomyelin in HPKs could be increased by GE. As shown previously, GE is not cytotoxic for human primary keratinocytes (HPKs) up to the tested concentration of 400 µg/mL [19]. As shown in Figure 1a, the overall ceramide content was unchanged by GE treatment. Glucosylceramides, which are a ceramide storage form and the precursors in keratinocytes, were ~1.6-fold increased after GE treatment (Figure 1b), whereas the other precursors, sphingomyelins, remained unchanged in GE treated HPKs (Figure 1c). Considering the individual ceramide subclasses, ceramides AS/AdS, which have sphingosine or dihydrosphingosine amide-linked to an α-hydroxy FA, were increased ~7-fold by GE (Figure 1d). Other ceramide subclasses detected in HPKs (ceramide NS, NdS, EOS, EOdS, NP and EOP) were present in equal quantities in treated and untreated cells (Figure 1e,f). 

### 2.2. Content of Ceramides and Glucosylceramides, as well as Protein Expression of ELOVL1, 4 and CerS3 in Lesional Skin of Psoriasis Patients compared to Healthy Skin

**Figure 2:**
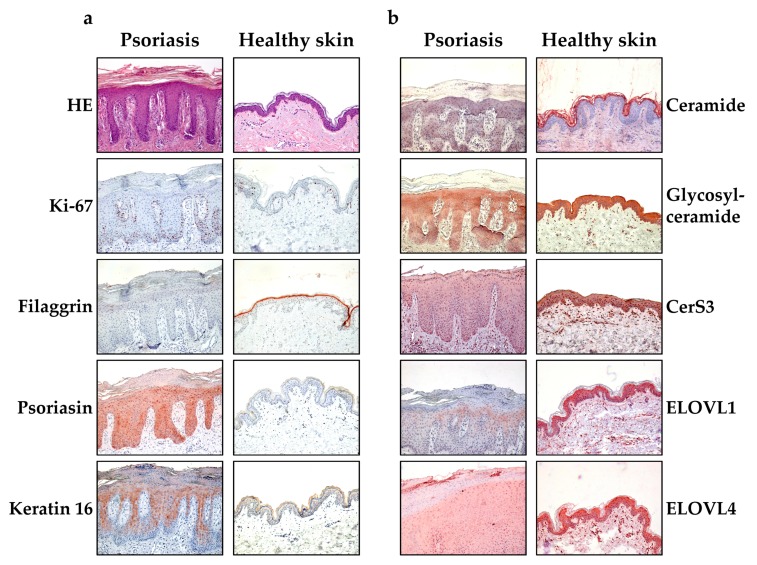
Expression of ceramides and enzymes for the generation of long chain free fatty acids (FA) ceramides in lesional skin of psoriasis patients. (**a**) Formalin- fixed and paraffin embedded tissue of lesional skin and healthy skin was HE stained. Furthermore Ki-67, filaggrin, keratin 16 and psoriasin was stained. (**b**) Formalin- fixed and paraffin embedded tissue of lesional skin and healthy skin was stained to detect ceramides, glucosylceramides, ceramide synthase 3 (CerS3) and elongases (ELOVL 1 and ELOVL4).

As GE increased the content of glucosylceramides and one ceramide subclass in HPKs, we looked at lipids that can be modulated by GE and enzymes, crucial for their synthesis in the lesional skin of patients with psoriasis and healthy volunteers (Figure 2). First, healthy skin tissue, as well as psoriatic skin, were HE stained to detect parakeratosis as a characteristic of psoriatic lesions. Then, the tissue was immunohistochemically stained to detect the proliferation marker Ki67 and the differentiation marker filaggrin. As expected, the proliferation was increased and the differentiation reduced in psoriasis tissue compared to healthy skin tissue. Furthermore, the expression of the psoriasis marker psoriasin (S100A7), and keratin 16, as marker of epidermal hyperproliferation, were increased in psoriatic skin (Figure 2a). In addition, the ceramide and glucosylceramide contents were reduced in psoriatic lesions compared to healthy skin (Figure 2b). Our staining showed a reduced protein expression of CerS3, ELOVL1 and ELOVL4 in psoriatic lesions compared to healthy skin (Figure 2b). These enzymes are crucial for the generation of very long chain ceramides [20]. The negative control showed no staining in healthy skin tissue or psoriasis (data not shown).

### 2.3. CerS3 and ELOVL4 Expression in GE treated Primary Keratinocytes as well as Psoriasis-Like Keratinocytes

**Figure 3:**
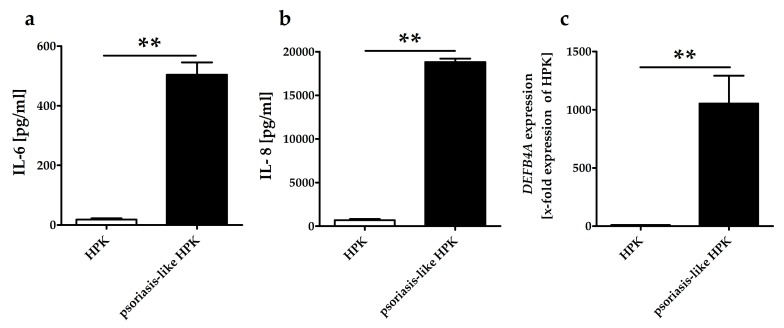
Characteristics of psoriasis-like HPKs compared to HPKs; (**a**) HPKs were incubated with psoriasis cytokines (IL-17, IL-22, TNF-α and INF-γ) for 24 h and the pro-inflammatory cytokine IL-6 was measured with an ELISA. (**b**) HPKs were incubated with psoriasis cytokines (IL-17, IL-22, TNF-α and INF-γ, 20 ng each) for 24 h and the chemokine IL-8 was measured with an ELISA (**c**) HPKs were incubated with psoriasis cytokines (IL-17, IL-22, TNF-α and INF-γ, 20 ng each) for 24 h, then RNA was isolated, reverse transcribed into cDNA and the gene expression of DEFB4A was measured in an qPCR reaction. Gene expression in relation to the control was calculated using the comparative C_T_ method from Schmittgen and Livak (see materials and methods). Data are presented as mean ± SD, *n* = 3, ** *p* ≤ 0.01.

In this study, HPKs were treated with IFN-γ, IL-17A, IL-22 and TNF-α, to generate a psoriasis-like phenotype. We established psoriasis-like keratinocytes, because the isolation of psoriatic keratinocytes from psoriasis lesions is very difficult. Leigh and colleagues found that the establishment of psoriatic keratinocyte cultures was only successful in 10% of cases [21] and psoriasis keratinocytes lose their specific psoriasis-characteristics in case they are no longer stimulated with psoriasis-cytokines. The psoriasis-like phenotype of stimulated HPKs was determined by analyzing the expression of pro-inflammatory cytokines and the expression of the psoriasis marker β-defensin 2 (DEFB4A). Psoriasis-like HPKs showed an increased protein expression of the psoriasis-associated pro-inflammatory cytokine IL-6 and the chemokine IL-8 (Figure 3a,b). The gene expression of the antimicrobial peptide and psoriasis marker DEFB4A was also augmented (Figure 3c). This proves that the psoriasis-like HPKs behave very similar to keratinocytes from psoriatic lesions.

**Figure 4:**
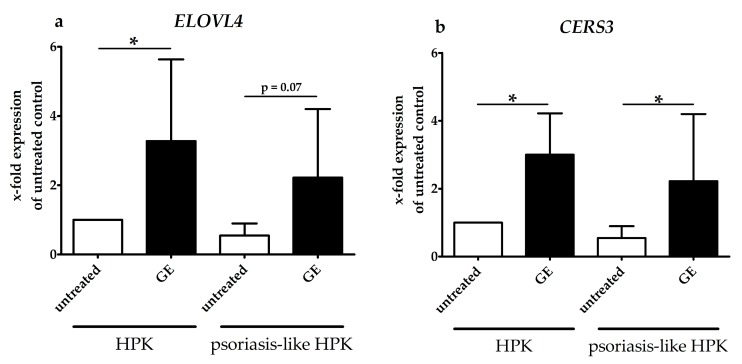
Expression of *ELOVL4* and *CERS3* in HPKs and psoriasis-like HPKs. (**a**) HPKs and psoriasis-like HPKs were incubated with GE (200 µg/mL) for 24 h. Total RNA was extracted and qPCR was performed to determine the *ELOVL4* gene expression level. To generate psoriasis-like HPKs, HPKs were incubated with GE (200 µg/mL) and after 2 h, stimulated with IL-17, IL-22, TNF-and IFN-γ (20 ng each) for 24 h. (**b**) HPKs and psoriasis-like HPKs were incubated with GE (200 µg/mL) for 24 h. Total RNA was extracted and qPCR was performed to determine the *CERS3* gene expression level. Gene expression in relation to the control was calculated using the comparative C_T_ method from Schmittgen and Livak (see materials and methods). Data are presented as mean ± SD, *n* = 3, * *p* ≤ 0.05.

We incubated normal HPKs and psoriasis-HPKs with GE and determined the CERS3 and ELOVL4 expression. GE did not show cytotoxic effects in HPKs or in psoriasis-like HPKs in the applied concentration of 200 µg/mL (data not shown). As shown in Figure 4a, GE increased the ELOVL4 expression in HPKs. While the expression level of ELOVL4 was reduced after cytokine treatment in psoriasis-like HPKs compared to normal HPKs, the incubation with GE partly rescued this effect. However, it reached only a borderline significance (*p* = 0.07). We then analyzed the effect of GE treatment on CERS3 expression in HPKs and psoriasis-like HPKs. GE significantly increased the CERS3 expression in GE treated HPKs and psoriasis-like HPKs compared to untreated cells (Figure 4b).

## 3. Discussion

Ceramides are generated from a sphingoid base and a fatty acyl-CoA. Fatty acyl-CoAs with carbon chains up to C16 can be synthesized by a FA synthase complex. However, for the generation of longer FAs, one of the seven ELOVL enzymes is required [22]. Furthermore, synthesis of ceramides with long chain FA requires CerS3, because CerS has different affinities to FA acyl-CoAs. CerS3 has a preference for C26 and C28 acyl-CoAs [23] and is especially highly expressed in the skin. The knockdown of ELOVL1, 4 or CerS3 in mice results in a lethal phenotype [4], because these mice are unable to build a functional skin barrier [24,25]. 

We have shown here that GE increases the synthesis of glucosylceramides and the ceramides AS/AdS in HPKs. This is consistent with the effects of GE on healthy skin, which increased the lipid content in vivo, as shown with bioengineering methods [19]. The reasons for the specific effect of GE on these ceramide subclasses are unknown. The information in the literature is also mixed concerning the role of the ceramides AS/AdS in skin diseases. It has been shown that in the skin of psoriasis and atopic dermatitis patients, ceramide AS is increased compared to healthy skin [10]. In contrast, another paper described a reduction in ceramide AS in nonlesional skin from allergic contact dermatitis patients, along with reduced barrier recovery rate after acute disruption by tape stripping [26].

GE also increased the abundance of glucosylceramides in HPKs. This effect could be beneficial, as the glucosylceramide depletion in basal keratinocytes affects keratinocyte maturation and hampers cornification and wound re-epithelialization [27]. In addition, as glucosylceramides are direct precursors of ceramides, it would be interesting in the future to verify whether these increased glucosylceramides will be converted into ceramides in psoriatic SC. Although the enzyme glucosylceramide-β-glucosidase (GBA), that hydrolyzes glycosylceramides into ceramides, is decreased in non-lesional psoriatic skin, more GBA is present in lesional psoriatic skin. This is probably a response to an altered water barrier [28]. Thus, the generation of more glucosylceramides in keratinocytes by treatment with GE might potentially lead to more ceramides in the SC, which would be beneficial to the patients with psoriasis to maintain relapse-free intervals.

We have shown in previous experiments that GE treatment has no effect on sphingomyelinase activity [19]. As sphingomyelin was not altered in GE-treated HPKs, it appears that sphingomyelin synthase was not affected by GE either. 

Previous studies have reported the different expression of specific ceramide subclasses in psoriasis, as well as a difference in FA chain length. In uninvolved skin from psoriatic patients, 223 transcripts were dysregulated compared to healthy skin. These genes can be attributed to various metabolic pathways, including the lipid metabolism [7]. ELOVL3 was one of the most downregulated genes in psoriasis patients. ELOVL3 mainly uses C18-C24 saturated and monounsaturated FAs as a substrate [4], whereas ELOVL4 expression that is upregulated by GE leads to C24 and C26 acyl chains in ceramides [29]. In addition, SPT is a critical enzyme in ceramide biosynthesis, that is significantly reduced in psoriatic lesions [30]. To assess the role of ceramides in the skin, Nakajima et al. generated mice with decreased ceramide levels in the epidermis, by knocking down serine palmitoyltransferase (SPT) [31]. Spt -/- mice showed an increased TEWL, an impaired barrier function and hyperkeratosis of the skin. The skin lesions showed an increased expression of psoriasis-associated genes such as IL17A, IL22 and DEFB4A. It was concluded that ceramide deficiency in the epidermis induces psoriasis-like lesions in mice. 

In accordance with our data, Tawada et al. have shown that pro-inflammatory cytokines, i.e., IFN-γ, reduce ELOVL4 gene expression in HPKs [16]. Furthermore, it was demonstrated that IL-17A and TNF-α had no effect on CERS3 and ELOVL4 expression in HPKs. However, as psoriasis is an IL-17A dominated disease, we added IL-17A, IL-22, TNF-α and IFN-γ to our psoriasis-like HPKs. We could show that a combination of IL-17A, TNF-α and IL-22 only weakly reduced the expression of *ELOVL4*. This effect could be increased by the addition of IFN-γ. We found that in HPKs, the cytokine mix with INF-γ reduces *ELOVL4,* but not *ELOVL1* expression (data not shown). GE could at least in part rescue this effect on *ELOVL4*. In addition, GE increased the *CERS3* expression in both healthy and in psoriasis-like HPKs.

Thus, GE clearly modulates sphingolipid synthesis in HPKs. Interestingly, it has been shown, more than 20 years ago, that an extract from the herbal bitter drug *Simarouba amara*, a tree from the rainforests of Southern America, increases the ceramide NP and EOH concentration in HPKs [32]. Similarly, Chon et al. have shown that the ceramide concentration in HPKs can also be increased by oat oil. The authors claimed that this might be mediated by activation of the PPARα and PPARβ/δ pathways [33]. These pathways are well-known for their capacity to improve the skin barrier by regulating epidermal lipid synthesis, lipid trafficking and keratinocyte differentiation [34,35]. The used oat oil had a high lipid content (3–18%), enriched with unsaturated FAs. It increased the expression of ELOVL4 that was partly reversed by PPARβ/δ antagonist treatment [33]. Strawberry seed extract with the polyphenol tiliroside as major component enhances the expression of PPARα, eventually leading to an increase of the total ceramide content and CERS3 expression. Specifically, the level of the ceramides NS and NdS was increased [36]. Very recently, it has been shown by the lipidomics profiling of human immortalized keratinocytes that the pentacyclic triterpene betulin differentially regulates the content of 440 lipid species, including increased ceramides [37].

The effects of these plant extracts on CERS3 and ELOVL4 expression in HPKs are comparable to the effect of GE that we described here. However, both oat oil and strawberry seed extract were only tested on HPKs and healthy skin models, but not in an inflammatory environment. In our psoriasis-like HPKs, GE also increased the ELOVL4 expression. Stimulating the expression of ELOVL1 or 4 and CERS3 might be beneficial for psoriasis patients with an increased IFN-γ production [17].

## 4. Materials and Methods 

### 4.1. Antibodies and Reagents

The following antibodies and dilutions were used for immunohistochemical stainings: polyclonal rabbit-anti-human CerS3 antibody (Antikörper-online, Aachen, Germany; 1:50), anti- ELOVL1 antibody Elabscience, Houston, TX, USA, 1:50), anti-ELOVL4 antibody (Abcam, Berlin, Germany, 1:50), anti-keratin 16 antibody (Santa Cruz, Heidelberg, Germany, 1:50), anti-Ki-67 antibody (DCS, Hamburg, Germany, 1:200), anti-filaggrin antibody (Santa Cruz, Heidelberg, Germany, 1:1000), anti-psoriasin antibody anti-ceramide antibody (Glycobiotech, Kükels, Germany, 1:25), anti-glucosylceramide antibody (Glycobiotech, Kükels, Germany, 1:50). The secondary antibody multi-link-biotin (Agilent-Dako, Hamburg, Germany, 1:200), the streptavidin-HRP-label (BioGenex, Fremont, IA, USA) and the AEC-substrate (MyBioSource, San Diego, CA, USA) were used according to the manufacturer’s instruction. IL-22, IL-17A, TNF- α, and IFN-γ were from Peprotech (Rocky Hill, CT, USA).

### 4.2. Preparation of Yellow Gentian Root High Pressure Ethanol (HPE) Extract

This procedure was already described and performed by Flavex Naturextrakte GmbH, 66780 Rehlingen, Germany [19]. In brief, whole gentian root was purchased from a Bavarian farm specialized in root drugs (Berghof Kräuter GmbH, Heilsbronn, Germany). The root was passed through a cutting mill with a 3 mm sieve (Pallmann Maschinenfabrik GmbH, Zweibrücken, Germany). The powder was filled into a high-pressure extractor and percolated with 8 kg of 96% (*v*/*v*) ethanol at 110 bar/60 °C per kg feedstock, to give the crude HPE (high pressure ethanol) extract. The major ethanol amount was removed by vacuum distillation, in order to adjust the final spissum extract to 50% dry matter, according to a drug/extract ratio of about 2.5/1. HPLC-UV chromatogram of yellow gentian root CO_2_-extract. The detection was at 232 nm: peak identifications (concentrations) are: loganic acid (3.1%), swertiamarin (0.79%), gentiopicroside (12.3%), loganin (0.41%), and amarogentin (0.05%).

### 4.3. Cell Culture

HPKs were prepared from reduction surgery of adult skin obtained from a dermatological surgery (approved by the ethics committee of the University Medical Center Freiburg (Certificate No EK432/18), and cultured according to the method of Rheinwald and Green [38] in DermaLife medium (Cell System, Goisdorf, Germany). All cells were cultured at 37 °C in a humidified atmosphere with 5% CO_2_. To generate psoriasis-like HPKs, HPKs were incubated with psoriasis cytokines (IL-17, IL-22, TNF-α and INF-γ, 20 ng each) for 24 h.

### 4.4. RT-PCR

The mRNA expression levels of ELOVL1, ELOVL4, CERS3, and β-defensin were measured by real-time qRT-PCR. Total RNA was extracted from HPKs using the trizol methode. After extraction, 1 µg of total RNA was reverse transcribed using iScript cDNA Synthesis Kit (BIO-RAD #1708890), to obtain cDNA. The real time qRT-PCR reaction was performed using a SYBR Green kit (Thermo Maxima SYBR Green qPCR Master Mix). The used primers were the following: 

hu ELOVL1: 5′-TGGCACTCTCCCTCTACATTGTCTA-3′ forward and 5′-TGAACTTGGAGAAGAGGAAGAGC-3′ reverse; hu CERS3: 5′-GCGGTTAACAAGTGGTGAAACA-3′ forward and 5′-TTCCTTCAGAGACCCACCCT-3′ reverse; hu DEFB4: 5′-ACCACCAAAAACACCTGGAAG-3′ forward and 5′-ACCAGGGACCAGGACCTTTA-3′ reverse; hu ACTB: 5′-CACTGTCGAGTCGCGTCC-3′ forward and 5′-TCATCCATGGCGAACTGGTG-3′ reverse. The relative gene expression was determined using the comparative C_T_ method [39], with ACTB as the internal control. 

### 4.5. Immunohistochemical Staining

2 µm sections of paraffin-embedded skin explants from either healthy skin tissue or psoriatic lesions (*n* = 3) were stained with the above listed primary and secondary antibodies using the LSAB method and photographed at 100× magnification. 

### 4.6. Lipid Analysis

Lipid levels were analyzed by high performance thin layer chromatography (HPTLC) [40]. HPKs were cultured in Dermalife in T 75 flasks. At subconfluence, the cells were treated with GE (200 µg/mL) in Dermalife, every second day for 1 week. Then, cells were detached, washed with PBS, dried and stored at −80 °C, until further analysis was performed. 

For lipid extraction, cells were resuspended in 3.6 mL chloroform/methanol 2:1 (*v*/*v*), sonicated for 10 min, and shaken for 1.5 h. The organic solvents were removed and the cells were shaken again with an additional 1.5 mL of chloroform/methanol 2:1 (*v*/*v*) for 1.5 h. The lipid extracts were combined, centrifuged, the supernatant was filtered, concentrated under a stream of nitrogen, dried under reduced pressure overnight, and stored at −20 °C.

A HPTLC analysis was performed on silica gel 60 HPTLC plates (20 × 10 cm^2^, Merck, Darmstadt, Germany). Lipids were dissolved in 200 μL chloroform/methanol 2:1 (*v/v*) and applied on the plate under a stream of nitrogen using Linomat 5 (Camag, Muttenz, Switzerland), along with standard lipids mixtures corresponding to the composition of human epidermal lipids (Pullmannova et al. 2014). The lipids were developed in an automatic developing chamber ADC 2 (Camag, Muttenz, Switzerland) with controlled humidity (33–36% RH) and temperature (25–27 °C). Ceramides were separated by developing twice (to 85 mm, 60 mm respectively) with chloroform/methanol/acetic acid 190:9:1.5 (*v*/*v*/*v*). The ceramide precursors were separated by developing once (to 85 mm) in chloroform/methanol/acetic acid/water 66:25:6:3 (*v*/*v*/*v*/*v*). To visualize the lipids, plates were dipped in a derivatization reagent (7.5% CuSO_4_, 8% H_3_PO_4_, and 10% methanol in water) for 10 s and heated at 160 °C for 20 min. Lipids were quantified by TLC scanner 3 and VisionCats software (Camag, Muttenz, Switzerland).

### 4.7. IL-6 and IL-8 ELISA

IL-6 and IL-8 concentrations in the supernatants were analyzed by a high sensitivity IL-6 and IL-8 ELISA (BD, San Jose, CA, USA), according to the manufacturer’s protocol. Data were expressed as mean ± SD of three independent experiments.

### 4.8. Statistical Analysis

The in vitro data were analyzed using the unpaired Student *t*-test (two-tailed). Statistical significance was established at * *p* ≤ 0.05. Data are expressed as mean ± SD of at least three independent experiments.

## 5. Conclusions

The skin of patients with psoriasis, both lesional and unaffected, show barrier abnormalities with fewer lipids. The topical application of ceramides or compounds that increase ceramide synthesis may improve skin barrier function [31]. Our findings suggest that topical treatment with GE, which partly rescues the *ELOVL4* downregulation in psoriasis-like HPKs and increases the *CERS3* expression, might contribute to the prevention and/or treatment of psoriasis.

## Abbreviations

CERS	Ceramide synthase
ELOVL	Elongase
GBA	Glucosylceramide-β-glucosidase
GE	*Gentiana lutea* extract
HPKs	Human primary keratinocytes
SC	Stratum corneum
SPT	Serine palmitoyltransferase
